# Detection of bacterial DNA by in situ hybridization in patients with decompensated liver cirrhosis

**DOI:** 10.1186/s12876-017-0664-z

**Published:** 2017-10-17

**Authors:** Shingo Usui, Hirotoshi Ebinuma, Po-Sung Chu, Nobuhiro Nakamoto, Yoshiyuki Yamagishi, Hidetsugu Saito, Takanori Kanai

**Affiliations:** 1 Division of Gastroenterology and Hepatology, Department of Internal Medicine, Keio University School of Medicine, 35 Shinanomachi, Shinjuku-ku, Tokyo, 160-8582 Japan; 2 Department of Internal Medicine, International University of Health and Welfare Mita Hospital, 1-4-3 Mita, Minato-ku, Tokyo, 108-8329 Japan; 3 Department of Internal Medicine, Tokyo Dental College Suidobashi Hospital, 2-9-18 Misakicho, Chiyoda-ku, Tokyo, 101-0061 Japan; 4 Faculty of Pharmacy, Keio University, 1-5-30 Shiba-kohen, Minato-ku, Tokyo, 105-8512 Japan

**Keywords:** Spontaneous bacterial peritonitis, Bacterial translocation, Liver cirrhosis, In situ hybridization, Blood culture

## Abstract

**Background:**

Spontaneous bacterial peritonitis (SBP) is often difficult to diagnose because bacteria in ascites cannot be detected accurately by conventional culture. In situ hybridization (ISH) was previously developed for rapid detection of genes from bacteria phagocytized by neutrophils. SBP may develop after bacteria enter into the systemic circulation following bacterial translocation. Therefore, we performed ISH to identify bacteria in blood samples collected from patients with decompensated liver cirrhosis (LC).

**Methods:**

In this retrospective study, peripheral blood samples were collected from 60 patients with decompensated LC, and bacteria were detected by both blood culture and ISH. Moreover, 35 patients underwent paracentesis for diagnosis of SBP.

**Results:**

Eight of 35 patients were diagnosed with SBP by polymorphonuclear neutrophil counts, and one patient was diagnosed with bacterascites. Seven of the nine patients showed positive results for ISH, whereas bacteria were detected in only two cases by blood culture. Thirty-seven of 60 cases (62%) showed positive results for ISH, whereas only six samples (10%) were positive by blood culture analysis. Compared with the 23 cases of negative ISH, the 37 cases of positive ISH showed a higher frequency of fever, higher Child-Pugh scores, and lower albumin levels.

**Conclusions:**

Detection of bacteria by ISH suggested that bacterial translocation, which cannot be proven by conventional culture, occurred in these patients, and that ISH could be helpful for the early diagnosis of some types of infection and prevention of SBP in these patients.

## Background

Patients with decompensated liver cirrhosis (LC) often suffer from various complications, such as hepatic encephalopathy, jaundice, gastroesophageal varices, and ascites. Spontaneous bacterial peritonitis (SBP), which was first reported by Conn and Fessle in 1971, is associated with a poor prognosis [1–4]. Early diagnosis and antibiotic treatment are necessary for management of SBP [4, 5]. However, it is difficult to diagnose SBP because bacteria in ascites or blood cannot be detected accurately by conventional culture in a timely manner. Polymorphonuclear neutrophil (PMN) counts in ascites are useful for diagnosis of SBP, and empirical antibiotic therapy should be started immediately after the diagnosis of SBP without knowledge of the causative bacteria [5].

The use of in situ hybridization (ISH) for detection of genes from bacteria phagocytized by neutrophils in ascites may have applications in the diagnosis of SBP [6]. ISH was developed to detect bacteria rapidly in patients with suspected bacterial infection [7] and can be applied to identify bacterial genes in neutrophils directly under a microscope after collection of blood samples from patients. Enomoto et al. reported that ISH is highly sensitive for detection of bacteria in ascites from patients with SBP [6]. Indeed, SBP may develop following bacterial translocation, during which enteric bacteria cross the intestinal epithelial cells, reach mesenteric lymph nodes [8], and enter into the systemic circulation [9]. Therefore, we hypothesized that patients with SBP may already exhibit systemic bacterial inflammation.

Accordingly, in this study, we attempted to detect bacteria in blood samples from patients with decompensated LC by ISH, and evaluated the utility of ISH compared with conventional blood culture in these patients.

## Methods

### Patients

**Fig. 1:**
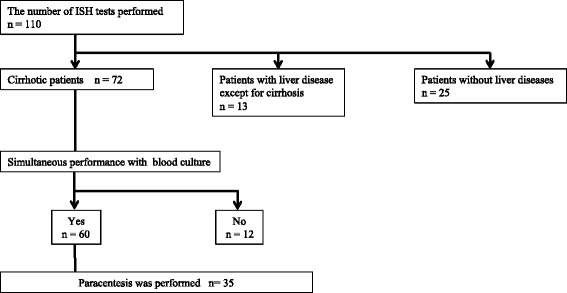
Algorithm for cases performed by ISH. We performed ISH 110 times for 94 cases. Twenty-five cases were patients without liver diseases, and 13 cases did not exhibit cirrhosis. Twelve of 72 patients with liver cirrhosis were examined with ISH only; thus, 60 samples were included in this study

Patients admitted to Keio University Hospital from April 2008 to March 2011 and who underwent ISH were included in this retrospective study. We performed ISH a total of 110 times in 94 patients. Twenty-five cases were patients without liver disease, and 13 cases were patients without cirrhosis. Sixty of 72 patients had ascites and were diagnosed with decompensated LC. Blood samples from 60 patients diagnosed with decompensated LC were simultaneously examined by ISH and blood culture. Moreover, 35 patients with mild or severe ascites underwent paracentesis to diagnose SBP by PMN counts of no less than 250/μL in ascitic samples, and ascitic samples were investigated with the blood samples simultaneously (Fig. 1). This study was approved by the ethics committee of our university hospital.

### ISH

ISH was performed using a commercial kit provided by Fuso Pharmaceuticals (Osaka, Japan). In ISH, the probes used to detect bacterial genomes for *Staphylococcus aureus* (SA), *S. epidermidis* (SE), *Pseudomonas aeruginosa* (PA), *Enterococcus faecalis* (EF), and a group of enterobacteria (*Escherichia coli, Enterobacter cloacae*, and *Klebsiella pneumoniae* [EK]) were previously established [7]. These pathogens are frequent causes of bacterial infections in patients with SBP [4, 10].

ISH was performed using blood samples from patients. Briefly, heparinized blood samples from patients were centrifuged (150×*g* for 10 min); leukocytes were collected after hemolysis with hypotonic buffer and resuspended in phosphate-buffered saline (PBS) at a concentration of 5 × 10^4^ cells/μL. A sample of the cellular suspension (5–10 μL) was spread onto a glass slide and air-dried. The slide was fixed in Carnoy’s solution for 20 min and permeabilized; bacterial DNA was denatured. Digoxygenin (Dig)-labeled probes and anti-Dig-alkaline phosphatase (ALP) were used for detection. Intracellular Dig-labeled hybridized signals were detected by anti-Dig-ALP. To visualize the signals, color development was achieved using nitro blue tetrazolium (NBT)-5-bromo-4-chloro-3-indolyl phosphate (BCIP) as a substrate for ALP. Positive signals in the cell cytoplasm were observed under a microscope. Blue-colored dots in neutrophils, representing phagocytic bacteria, were assumed to represent a positive result.

### Blood and ascitic cultures

All blood and ascitic samples were collected into both aerobic and anaerobic blood culture bottles. Blood and ascitic culture bottles were sent to the reference microbiology laboratory of our hospital for processing. The blood culture system BD Bactec FX (Nippon Becton Dickinson, Tokyo, Japan) was used for the detection of pathogens. Samples were incubated at 37 °C for 5 days. Isolates of microorganisms were identified using a Bactec FX system.

### Statistical analysis

Comparisons of the frequencies between the two groups were analyzed by McNemar’s tests. Baseline data for patients with LC were statistically evaluated. Variables were expressed as the mean ± standard deviation (SD), and differences between two groups were evaluated by Student’s t-tests, and three groups by ANOVA.

## Results

### Patient background

Table 1 shows the characteristics of the 60 patients included in this study. ISH and conventional blood cultures were performed in 60 patients with ascites out of 72 decompensated LC patients. The median age was 62 years, and the patients were predominantly male (male:female = 35:25). The causes of cirrhosis were hepatitis B virus infection (*n* = 4), hepatitis C virus infection (*n* = 18), alcohol (*n* = 21), and other factors (*n* = 17). All patients had ascites, and their Child-Pugh scores were no less than 8. The Child-Pugh classifications were B and C, and the mean score was 9.97 ± 1.55. The median total bilirubin value was 2.85 mg/dL, and the maximum value was 29.2 mg/dL. The mean albumin and creatinine values were 2.41 ± 0.47 mg/dL and 1.18 ± 0.71 g/dL, respectively. The median Model For End-Stage Liver Disease (MELD) score was 15. Patients with diabetes were those who were diagnosed as such by fasting blood glucose or oral glucose tolerance test and treated with insulin or hypoglycemic drugs.

**Table 1:** Baseline characteristics of patients with cirrhosis who underwent ISH

	Mean ± SD or median value (range)
Age (years)	62 (29–84)
Sex (male/female)	35/25
Etiology (HBV/HCV/alcohol/others)	4/18/21/17
Child-Pugh classification (B/C)	23/37
Child-Pugh score	9.97 ± 1.55
Total bilirubin (mg/dL)	2.85 (0.3–29.2)
Albumin (g/dL)	2.41 ± 0.47
Prothrombin time (INR)	1.38 (0.97–2.38)
Creatinine (mg/dL)	1.18 ± 0.71
White blood cell counts (/μL)	6350 (2000–27,800)
Platelet counts (× 10^3^/μL)	78.5 (20–760)
C-reactive protein (mg/dL)	2.42 (0.12–15.53)
MELD score	15 (7–34)
Esophageal varices (+/−)	36/24
Hepatocellular carcinoma (+/−)	19/41
Abdominal pain (+/−)	4/56
Diabetes mellitus (+/−)	17/43

Valuables are the mean ± standard deviations (SDs) or median (range)

HBV: hepatitis B virus, HCV: hepatitis virus

### Comparison between ISH and blood cultures in patients undergoing paracentesis

**Fig. 2:**
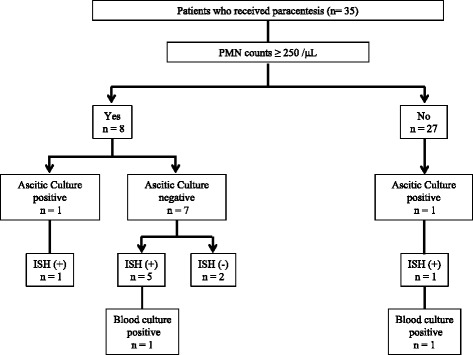
Algorithm of patients who received paracentesis. Eight of 35 patients were diagnosed with spontaneous bacterial peritonitis (SBP). Six cases of SBP (75.0%) were positive for bacteremia by ISH, but only one patient (12.5%) was positively identified by blood bottle culture

Eight cases were diagnosed with SBP based on PMN counts. The diagnosis of SBP was based on elevated PMN counts (≥ 250/μL) in 35 cases undergoing paracentesis (Fig. 2). ISH results were positive in six of eight SBP cases, whereas only one case was positive for blood culture. One patient who was positive for ascitic culture was also positive for ISH but negative for blood culture. In seven ascitic culture-negative cases, five were positive for ISH, and one was positive for blood culture. One patient was diagnosed with bacterasites and showed positive results for both ISH and blood culture. Of note, 14 of 27 patients who were not diagnosed with SBP by PMN counts of ascites were positive for ISH, and three out of 14 patients later proved to be positive for blood culture, suggesting that ISH might be useful to diagnose bacterial infection in decompensated LC patients earlier than conventional ways. We did not find any cases who were negative for ISH but positive for ascitic cultures or blood cultures.

### Differences in detection rates between ISH and blood culture

Of the 60 cases who underwent both ISH and blood culture, six were positive for bacteremia by blood culture (10.0%), whereas 37 were positive by ISH (61.7%; *p* < 0.001; Table 2). The rate of positive detection by ISH was significantly higher than that by blood culture. All positive cases by blood culture were positively detected by ISH, and no cases were negative for ISH but positive for blood culture.

**Table 2:** Differences in detection rates between blood culture and ISH

		Blood culture		Total
		Positive	Negative	
ISH	Positive	6 (10.0%)	31 (51.7%)	37 (61.7%)
	Negative	0 (0%)	23 (38.3%)	23 (38.3%)
Total		6 (10.0%)	54 (90.0%)	60 (100%)

### Clinical features and detection of ISH

Thirty of the 60 patients had fever. Among the patients with fever, 23 cases (76.7%) were positive for ISH analysis, and six (20.0%) were positive for blood cultures. Among the patients without fever, 14 cases (46.7%) were positive for ISH analysis, but none was positive for blood cultures. In 47 C-reactive protein (CRP)-positive cases (≥ 1 mg/dL), 30 (63.8%) and four (8.5%) cases were positive for ISH analysis and blood cultures, respectively. The rates of positive results between ISH and blood cultures differed significantly, suggesting that ISH may detect bacteria more sensitively than conventional blood culture (Table 3). Among 24 patients with antibiotic use, oral or intravenous antibiotic treatment was given to 22 patients for confirmed or suspected bacterial infections with unidentified origin. Two patients received an oral aminoglycoside bactericidal antibiotic called kanamycin for prevention of SBP. It is possible that using antibiotics in these patients possibly affected the sensitivity of blood culture.

**Table 3:** Positive detection rates by ISH and blood cultures according to clinical manifestations

	No	ISH positive	Blood culture Positive	*P* value
Total	60	37 (61.7%)	6 (10.0%)	< 0.001
Fever	30	23 (76.7%)	6 (20.0%)	< 0.001
No fever	30	14 (46.7%)	0 (0.0%)	–
CRP ≥ 1 mg/dL	47	30 (63.8%)	4 (8.6%)	< 0.001
CRP < 1 mg/dL	13	7 (53.8%)	2 (15.4%)	0.0253
Antibiotic use	24	15 (62.5%)	2 (8.3%)	< 0.001

Statistical significance was determined by McNemar’s test

Moreover, we compared the clinical characteristics between ISH/blood culture positive (+/+), ISH positive-blood culture negative (+/−), and both negative (−/−) patients (Table 4). There were significant differences in the frequency of clinical symptom of fever between the three groups (*p* < 0.05), suggesting that ISH or the combination of ISH and blood culture is useful for the early diagnosis of bacterial infection. Further study in a larger sample size is needed to validate the results.

**Table 4:** Comparison of clinical manifestations among positive (+/+), ISH positive-blood culture negative (+/−), and fully negative (−/−) patients

ISH/blood culture	(+/+)	(+/−)	(−/−)	*P* value
No.	6	31	23	
Fever (+)	6 (10 0%)	17 (55%)	7 (30%)	< 0.001
CRP (mg/dL)	3.40 ± 1.44	3.98 ± 0.63	2.92 ± 0.75	N.S.
Child-Pugh score	11.3 ± 0.6	10.1 ± 0.3	9.4 ± 0.3	0.020
MELD score	18.0 ± 2.6	17.4 ± 1.1	15.6 ± 1.3	N.S.
Total bilirubin (mg/dL)	7.62 ± 2.97	6.85 ± 1.30	4.34 ± 1.51	N.S.
Albumin (g/dL)	2.17 ± 0.19	2.33 ± 0.08	2.58 ± 0.10	N.S.(0.066)
Prothrombin time (INR)	1.49 ± 0.11	1.40 ± 0.05	1.38 ± 0.06	N.S
Platelet counts (× 10^3^/μL)	68.8 ± 45.2	99.7 ± 19.9	134.7 ± 23.1	N.S.
Diabetes mellitus (+)	4 (67%)	9 (29%)	4 (17%)	0.049
HCC (+)	4 (67%)	7 (23%)	8 (35%)	N.S.
Esophageal varix (+)	4 (67%)	18 (58%)	14 (61%)	N.S.

### Detection of bacterial groups by ISH

Table 5 shows the DNA probes used for detection of bacteria in ISH and the number of positive cases by each probe. The EK probe was positive in almost all cases (81%). Eight cases showed positive ISH results for the EK probe and another probe simultaneously.

**Table 5:** Bacterial strains detected by ISH

Bacteria	ISH probe	Number of positive cases
Escherichia coli		
Enterobacter cloacae	EK	30 (81.1%)
Klebsiella pneumoniae		
Enterococcus faecalis	EF	6 (16.2%)
Staphylococcus aureus	SA	5 (13.5%)
Pseudomonas aeruginosa	PA	4 (10.8%)
Staphylococcus epidermidis	SE	0 (0.0%)

## Discussion

In this study, we successfully employed ISH to detect pathogens in the blood from patients with decompensated LC in whom blood culture results were negative for bacterial infection. These findings suggested that bacterial translocation cannot be always detected by conventional blood culture because SBP is known to occur after bacterial translocation, defined as the passage of bacteria from the intestine or colon through the intestinal epithelial cells and entrapment in the mesenteric lymph nodes [8, 11]. After bacterial translocation, bacteria are thought to enter the systemic bloodstream and access ascitic fluid, which exhibits low bactericidal capacity [9, 12–14]. Bacterial translocation has been demonstrated in some studies in both human and animal models of LC [15, 16]. However, it is unclear how SBP develops from bacterial translocation because this event cannot be detected easily by conventional blood culture [11]. Such et al. reported that bacterial DNA can be detected simultaneously in blood and ascitic fluid [17, 18], using a polymerase chain reaction (PCR)-based method. Although this method may provide evidence of the relationship between bacterial translocation and SBP, no studies have compared PCR-based methods with blood culture. Therefore, we hypothesized that ISH could be applied to decompensated LC patients to detect bacteria and may be helpful for selecting patients who may have an infection earlier and determining the proper antibiotic to use if bacteria are present in the systemic bloodstream after bacterial translocation.

ISH was first developed to enable early diagnosis of sepsis within 1 day, using ISH of the bacterial genomes existing in neutrophils after phagocytosis. This method was reported to be four times as sensitive as blood culture for detection of bacteria in patients with sepsis [7]. The other advantages of the ISH method are that it can eliminate potential contamination and is not affected by antibiotic use, as it analyzes pathogens already captured into neutrophils by phagocytosis. Thus, the probability of false-positive results is low with this procedure. Enomoto et al. developed a new probe mixture, designated a global bacteria (GB) probe, which was capable of detecting all relevant bacterial strains. Using ISH, this probe showed positive results in 10 of 11 SBP cases and negative results in none of 40 non-SBP cases in ascites [6]. Bacteria causing SBP are frequently gram-negative rods, such as *Escherichia coli* and *K. pneumoniae*, or can be *Streptococcus* species [10]. Such et al. studied bacterial DNA to show that *Escherichia coli* were the most frequently identified bacteria [18]. This is consistent with our results demonstrating that *Enterobacteria* were frequently detected. Therefore, even five specific probes without the global bacteria probe may be useful for detection of bacteria in patients with decompensated LC. These results suggest that early detection of specific bacteria causing SBP and early therapeutic intervention using appropriate antibiotics are additional advantages of ISH for patients with LC and SBP. However, further studies are needed to confirm this assumption. Additionally, blood cultures beyond ISH tests are thought to be necessary for such patients because bacteria other than the five species probed by ISH may be detected and because drug susceptibility tests cannot be performed by ISH.

Interestingly, in our study, some clinical parameters were related to the results of ISH tests, including the presence of fever and Child-Pugh scores. These results may also be related to the occurrence of bacterial translocation. Cirera et al. detected enteric organisms increasingly from mesenteric lymph nodes in patients with or without cirrhosis according to the Child-Pugh score: 3.4% in A, 8.1% in B, and 30.8% in C [15]. Bacteria in neutrophils were detected in 47% of patients without fever and in 54% of patients whose CRP levels were below 1 mg/dL in our study. Surprisingly, bacterial translocation may already be present in asymptomatic patients with LC having ascites. Evans et al. reported that 3.5% of all outpatients with cirrhosis had SBP, and 1.9% of these patients had bacterascites [19]. Moreover, several studies have also demonstrated that more severe liver failure is associated with lower CRP levels [20, 21]. Administration of antibiotics may be considered to prevent further deterioration of sepsis or SBP in decompensated LC patients positive for ISH; these patients have no clinical symptoms at this point.

Recent reports have shown that serum albumin functions to maintain oncotic pressure and has immunomodulatory and antioxidant effects. Albumin infusions were found to reduce the incidence of renal failure and mortality in patients with SBP [22, 23]. Patients with positive results by ISH had higher Child-Pugh scores and showed a tendency of lower serum albumin levels (Table 4). If hypoalbuminemia indicates immunological deterioration in patients with decompensated LC, patients who are positive for ISH may be required to receive albumin infusions early in addition to antibiotics.

The limitations of this study include the small number of subjects. Obviously, a larger controlled study will be needed to validate the results and confirm the usefulness of ISH.

## Conclusions

In conclusion, bacterial translocation, which often occurs in ascitic patients with LC, cannot be proven by methods such as conventional blood cultures. ISH may be helpful to select patients who are suspected of having an infection, including SBP, and to manage treatment in a timely manner, although additional studies are required.

## Abbreviations

CRP: C-reactive protein
EF: *Enterococcus faecalis*
EK: a group of enterobacteria
HCC: hepatocellular carcinoma
ISH: in situ hybridization
LC: liver cirrhosis
MELD: Model For End-Stage Liver Disease
PA: *Pseudomonas aeruginosa*
PCR: polymerase chain reaction
PMN: polymorphonuclear neutrophil
SA: *Staphylococcus aureus*
SBP: spontaneous bacterial peritonitis
SE: *Staphylococcus epidermidis*
